# Cascading effects of belowground predators on plant communities are density‐dependent

**DOI:** 10.1002/ece3.1597

**Published:** 2015-09-12

**Authors:** Madhav Prakash Thakur, Martina Herrmann, Katja Steinauer, Saskia Rennoch, Simone Cesarz, Nico Eisenhauer

**Affiliations:** ^1^ German Centre for Integrative Biodiversity Research (iDiv) Halle‐Jena‐LeipzigDeutscher Platz 5e04103LeipzigGermany; ^2^ Institute of BiologyUniversity of LeipzigJohannisallee 2104103LeipzigGermany; ^3^ Institute of EcologyFriedrich Schiller Jena UniversityDornburger Str. 15907743JenaGermany

**Keywords:** Complementarity effects, interspecific competition, microbial community, N uptake, nitrification rates, soil food web, trophic cascade

## Abstract

Soil food webs comprise a multitude of trophic interactions that can affect the composition and productivity of plant communities. Belowground predators feeding on microbial grazers like Collembola could decelerate nutrient mineralization by reducing microbial turnover in the soil, which in turn could negatively influence plant growth. However, empirical evidences for the ecological significance of belowground predators on nutrient cycling and plant communities are scarce. Here, we manipulated predator density (*Hypoaspis aculeifer*: predatory mite) with equal densities of three Collembola species as a prey in four functionally dissimilar plant communities in experimental microcosms: grass monoculture (*Poa pratensis*), herb monoculture (*Rumex acetosa*), legume monoculture (*Trifolium pratense*), and all three species as a mixed plant community. Density manipulation of predators allowed us to test for density‐mediated effects of belowground predators on Collembola and lower trophic groups. We hypothesized that predator density will reduce Collembola population causing a decrease in nutrient mineralization and hence detrimentally affect plant growth. First, we found a density‐dependent population change in predators, that is, an increase in low‐density treatments, but a decrease in high‐density treatments. Second, prey suppression was lower at high predator density, which caused a shift in the soil microbial community by increasing the fungal: bacterial biomass ratio, and an increase of nitrification rates, particularly in legume monocultures. Despite the increase in nutrient mineralization, legume monocultures performed worse at high predator density. Further, individual grass shoot biomass decreased in monocultures, while it increased in mixed plant communities with increasing predator density, which coincided with elevated soil N uptake by grasses. As a consequence, high predator density significantly increased plant complementarity effects indicating a decrease in interspecific plant competition. These results highlight that belowground predators can relax interspecific plant competition by increasing nutrient mineralization through their density‐dependent cascading effects on detritivore and soil microbial communities.

## Introduction

Global projections of predator decline due to global change effects, such as climate warming or land‐use change (Estes et al. 2011), can trigger alterations of several ecosystem functions (Both et al. 2009; Zarnetske et al. 2012). Predators mainly contribute to ecosystem functions by regulating prey populations and subsequent cascading effects on the lower trophic groups and associated process rates. In plant–herbivore–predator systems, predators can indirectly enhance plant production by reducing herbivore populations (Schmitz et al. 2004; Terborgh et al. 2010). In microbe–detritivore–predator systems in soil, cascading effects of predators on soil microorganisms can also alter the performance of plant communities (Bardgett and Wardle 2010; Kulmatiski et al. 2014). For instance, detritivores help convert complex organic forms of nutrients into simpler forms and accelerate microbial turnover, which facilitate nutrient uptake of plants (Moore et al. 2003). Hence, predators of detritivores could potentially exert detrimental effects on plants by reducing the rates of nutrient cycling. However, the strength of such predator effects may vary among plant communities and plant species therein as plants may differ in their dependency on nutrient availability in the soil and in their association with soil microbial symbionts. Grass species, for example, depend largely on soil nutrient turnover by detritivores compared to legume species that have the ability of acquiring nitrogen from the atmosphere through the association with rhizobia (Eisenhauer and Scheu 2008; Jackson et al. 2008).

Nitrogen (N) availability in soil greatly depends on the mineralization process driven by soil microorganisms. Detritivores like Collembola regulate the microbial turnover and hence mineralization processes in soil by directly feeding on fungi and bacteria (Cragg and Bardgett 2001). Accordingly, microbial‐grazing Collembola have been shown to benefit plant communities (Kulmatiski et al. 2014), although the strength of those positive effects can differ among plant functional groups (Eisenhauer et al. 2011b). Notably, some soil microorganisms compete with plants for available N, while detritivores can relax this competition by regulating microbial population size (Cherif and Loreau 2009). Predator‐induced reduction in detritivore densities could enhance soil microbial population size, which implies an increased net demand for N by microorganisms (Woods et al. 1982). This, in turn, may enhance the competition between plants and microorganisms for N (Kuzyakov and Xu 2013).

Studies on predator‐induced effects on the lower trophic levels in soil have found inconsistent results (Wardle 2002). Mikola and Setälä (1998) reported that the presence of predatory nematodes had non‐significant effects on the microbial community, although predatory nematodes reduced the population of microbial‐feeding nematodes. Moreover, studies that manipulated predatory mites, common belowground top predators feeding on Collembola, oribatid mites, and larger nematodes (Ruiter et al. 1995), have found both positive and negative effects on N mineralization (reviewed in Wardle 2002). For instance, Hedlund and Ohrn (2000) reported positive effects of predatory mites on N mineralization rates after increased fungal colonization due to a predator‐induced decrease in Collembola densities. Predatory mite‐induced negative effects on N mineralization occurred in studies when microbial communities were unaffected by the reduction in microbial feeders (Wardle 2002). Recently,Thakur et al. (2014) showed that the presence of predatory mites can significantly increase nitrate availability in soil depending on prey community composition. They found that compensatory increases in enchytraeid densities, a common fungal grazer, increased nitrate availability in the presence of predatory mites and oribatid mites (the latter being another prey) (Thakur et al. 2014). Such results indicate that predators can alter prey community composition, that is, decreasing community evenness by favoring one prey over the other (Filip et al. 2014).

Shifts in the availability of N due to belowground trophic cascades may interact with the composition of the plant community. For instance, legumes can benefit neighboring nonlegume plants in mixed plant communities by fixing N from the atmosphere through symbiosis with rhizobia, increasing soil N availability, and thus relaxing the plant–microbe competition (Schmidt 1979). Contrarily, at higher N availability in soil, legumes acquire less N from the atmosphere (Jackson et al. 2008) and due to their poor root‐based foraging for nutrients in soil, neighboring plant species, such as grasses, have a competitive advantage over legumes (Eisenhauer and Scheu 2008; Skogen et al. 2011). As a consequence, soil predators could decrease the dominance of certain plant species like grasses and alter plant community composition by changing the size and composition of detritivore communities, soil microbial communities, and N availability. Recently, Kulmatiski et al. (2014) showed that positive cascading effects of belowground predators on plants were pronounced when predators suppressed belowground plant pathogens and herbivores, while plants still benefited when predators suppressed their mutualists, such as those increasing nutrient mineralization. However, previous studies have rarely considered how belowground predator‐induced suppression of microbial grazers may vary in the context of different plant communities and possibly cascade to plant performance (Laakso and Setälä 1999; Bardgett and Wardle 2010; Kulmatiski et al. 2014).

Here, we study the effects of predatory mites (as a predator of Collembola) on soil microbial communities and associated N dynamics in different plant communities with three initial predator densities (low, intermediate, and high). It is likely that different initial densities of predators and changes of predator densities over the course of an experiment may exert different predation pressures on prey. For instance, prey suppression and cascading effects may be higher when predator densities increase over the experimental period compared to decreases in predator densities (Schausberger and Walzer 2001; Levi et al. 2015). We hypothesize that predator density will alter microbial community composition with a negative effect on N mineralization due to the suppression of Collembola densities (Hypothesis 1). Further, we hypothesize that predator density will negatively affect nonlegume species in monoculture due to their higher dependence on nutrient turnover in soil than legumes (Hypothesis 2). Finally, we hypothesize that predator‐induced negative effects in the mixed plant community (containing both legume and nonlegume species) will be diluted due to legume‐induced benefits to neighboring plants through N fixation (Hypothesis 3).

## Methods

### Belowground predator‐prey fauna

The predator species used was *Hypoaspis aculeifer*, which is a common soil subsurface dwelling predatory mite from the Hypoaspididae family (Koehler 1999). They can feed on numerous prey items ranging from larger nematodes to Collembola and are also used as a bio‐control agent (Koehler 1999). Depending on the prey item and temperature, their developmental rate (from egg to adult) varies from as long as 30–33 days at 13°C to as short as 5 days at 28°C (Siepel 1994). At 18–20°C, close to the temperature range in this experiment (16–20°C), the developmental rate of *Hypoaspis aculeifer* is reported to range from 13 to 20 days (Siepel 1994). Predators were purchased from Schneckenprofi in Germany (http://www.schneckenprofi.de/). The three Collembola species *Folsomia candida, Proisotoma minuta,* and *Sinella curviseta* were used as a model detritivore community and prey for *H. aculeifer*. All three Collembola species were cultured with yeast at room temperature in the laboratory. Families of all three Collembola species are found in nearby grasslands (Sabais et al. 2011) together with the focal plant species (see below). These Collembola were reported to be microbial grazers (Hopkin 1997).

### Plant communities

Three grassland plant species were used to represent three plant functional groups: *Trifolium pratense* (legume species; common name: Red clover), *Poa pratensis* (grass species; common name: Kentucky bluegrass), and *Rumex acetosa* (herb species; common name: Common sorrel). All these species are common grassland species in Central Europe. We selected two nonlegume species based on their differences in spatial nutrient acquisition from soil (Ebeling et al. 2014), which could be due to differences in their root architecture (Veresoglou and Fitter 1984; Voesenek and Blom 1987). All plants were germinated in defaunated soil (described below) prior to the experiment and transplanted later into microcosms. The respective seeds were purchased from Rieger‐Hoffmann GmbH, Blaufelden‐Raboldshausen, Germany.

### Experimental set‐up and harvest

The experiment was carried out in PVC microcosms (height 10 cm; diameter 7 cm) filled with 300 g of defaunated soil from a grassland floodplain (Ebeling et al. 2014). The soil was sandy loam with pH of 8.1 and C:N ratio of 15.7 (Roscher et al. 2004). Prior to defaunation, the soil was sieved using a 2‐mm mesh in order to remove roots and stones. For defaunation, the soil was initially frozen at −20°C for 48 h and then thawed at room temperature for several days. This cycle was repeated twice. This method of defaunation has been used efficiently to remove soil fauna with a minimal effect on the microbial community (Poll et al. 2007). Nevertheless, to increase microbial colonization after defaunation, we incubated the soil in microcosms by adding 10 mL of tap water per day for 2 weeks (the same amount of water was added every day throughout the experiment). At the start of incubation, 500 mg of ^15 ^N‐labeled grass litters of *Lolium perenne* (30 atom% ^15^N) of ~1 mm of size were mixed on the top soil to measure nutrient assimilation by the plants. The experiment was performed at a defined day/night cycle with a controlled temperature (16 h light at 20°C and 8 h dark at 16°C).

Germinated plants (6 weeks of germination, height of 5–8 cm determined using a ruler) were carefully transplanted into the microcosms. We established three different monocultures for three species by planting three individuals per species into each microcosm. A mixed plant community was established with all three species together with one individual per species. Twenty days after transplanting seedlings, Collembola were added to the microcosms. We added 20 individuals per species, that is, in total 60 individuals of Collembola per microcosm. Collembola densities were in the range of field density Collembola (20,000 ind/m^2^) in the nearby grasslands (Eisenhauer et al. 2011a). Predatory mites were added 1 week after Collembola in three different densities: low (3 individuals), intermediate (9 individuals), and high (15 individuals). Hence, we established four plant communities crossed with three predator density treatments replicated five times (60 microcosms in total).

Microcosms were randomly arranged in five blocks to account for variations caused by light conditions and wind flow in the greenhouse chamber. All microcosms were kept in the same greenhouse chamber. The experiment ran for 8 weeks after the addition of predators in order to sufficiently capture developmental rates of predatory mites, and by this time, plants were also reaching maturity (14 weeks). At the harvest, we sampled soil cores for faunal extraction, microbial community estimation, and nitrification rate measurements. We measured plant species‐specific shoot biomass, community root biomass, and ^15^N concentration in shoot material. Plant species‐specific shoot biomass and plant community root biomass (thoroughly cleaned to remove sand attached to roots) were obtained after drying plants for 72 h at 70°C. Roots were recovered from soil cores used to extract animals and from the microcosms and later pooled together as a measure of community root biomass.

### Extraction of soil fauna

Soil cores (5 cm deep and 5 cm diameter) were taken from microcosms after the shoot material of plants had been removed. These soil cores were used to extract Collembola and predatory mites using heat extraction (Macfadyen 1961). This method requires a gradual heating of soil cores for 10 days from 25°C up to 50°C. Collembola and predatory mites were collected in glycol and were later transferred to 70% ethanol for preservation. Animals were counted using a dissecting microscope.

### Microbial community composition

Microbial community composition in soil was determined using phospholipid fatty acid (PLFA) composition as described in Frostegård et al. (1991) from the 5‐cm‐deep soil cores taken during the final harvest. We used individual PLFA markers to quantify the biomasses of specific microbial groups based on Ruess and Chamberlain (2010). We grouped twelve specific PLFA markers into three functional groups of soil microorganisms: gram‐positive bacteria, gram‐negative bacteria, and fungi. Gram‐positive bacteria included the following markers: i14:0, i15:0, a15:0, i16:0, i17:0, a17:0, i18:0, and a18:0. Gram‐negative bacteria included cy17:0 and cy19:0 PLFA markers, whereas fungal markers included 18:1*ω*9 and 18:2*ω*6,9. We used three indices to indicate microbial community structure: gram‐positive bacteria: gram‐negative bacteria ratio, fungi: bacteria ratio, and PCA axis 1 scores calculated using all twelve PLFA markers (see Data analysis). We used total PLFA as an indicator of living microbial biomass (Chung et al. 2007).

### Potential nitrification rates

Nitrification rates were measured to represent a part of the N mineralization process in soil. We used the shaken‐slurry method (Yao et al. 2011) to estimate soil nitrification rates at the final harvest. We took 15 g of fresh (5 cm deep) soil from the microcosms during the harvest and resuspended them in 100 mL of 1.5 mmol/L ammonium sulfate. Soil slurries were incubated at 22°C and constant agitation (90 rpm) for 40 h with six subsamples being taken during this period. Slurry subsamples were centrifuged and stored at −21°C until nitrate analysis was performed. Net potential nitrification rates (nmol ${\text{NO}}_{3}^{-}$ ‐N/g soil (dry weight)/h) were calculated from the rate of increase in ${\text{NO}}_{3}^{-}$ concentration over time in the slurry using linear regression.

### ^15^N measurement

^15^N concentrations in plant shoot material were determined in order to quantify nutrient acquisition by different plant species in response to the experimental treatments. A subsample of plant shoot material from each microcosm (pooled for monoculture and species‐specific samples from the mixed plant community) was grinded into powder of which ~3 mg were put into tin capsules. ^15^N/^14^N isotope ratios (*δ* ^15^N) were then determined using these tin capsules in a coupled system consisting of an elemental analyzer (NA 1500, Carlo Erba, Mailand) and a mass spectrometer (Delta C, Finnigan MAT, Bremen, Germany). We quantified ^15^N uptake of plants in their monoculture and in the mixed plant community as a product of individual plant biomass and *δ* ^15^N.

### Plant complementarity and selection effects

In order to compare the plant performance in the mixed plant community vs. monoculture plant communities and to investigate potential changes in plant community composition, we calculated complementarity and selection effects based on the additive partitioning approach proposed by Loreau and Hector (2001) using plant species‐specific shoot biomass data. The complementarity and selection effects were calculated for three predator density treatments using the formula (Loreau and Hector 2001):

Complementarity effect=N∗mean(ΔRY)∗mean (M)

Selection effect=N∗covariance(ΔRY, M)

where, *N *= number of species in the mixed plant community, ∆RY = deviation from expected relative yield (shoot biomass) of the particular species in the mixed plant community, and *M = *yield of the particular species (shoot biomass) in its monoculture. The details for calculating complementarity and selection effects are provided in Loreau and Hector (2001). Increasing complementarity effects are a representation of greater niche differentiation and facilitative interactions among species (decrease in interspecific competition), whereas an increase or decrease in selection effects indicates changing dominance of single species in total productivity (or performance) of a plant community (Loreau and Hector 2001; Wagg et al. 2011; Turnbull et al. 2012).

### Data analysis

In order to test initial predator density treatment effects on the final densities of predators at the end of experiment, we calculated the increase in predator density as proportional increase in predator density [(final density‐initial density)/(initial density)] and expressed as %. Proportional increase in predator density over the experiment period can be used to indicate the strength of density dependence in population change and intraspecific competition (Abrams 1998). For instance, if high‐density predator treatments were to show a proportional decline compared to low‐density treatment, this would infer negative density dependence in high‐density treatments (Hassell 1975; Vandermeer and Goldberg 2013). Further, we calculated predator: prey ratio using the densities of the predator and Collembola at the end of the experiment. The predator: prey ratio can be used as an index to indicate prey availability for predators (Murphy et al. 2012). Higher predator: prey ratio indicates a greater impact of predator on prey (Symondson et al. 2002; Murphy et al. 2012). We also calculated prey community evenness as Simpson evenness (∑*p* ^2^/S, where *p* is the proportion of species and *S* is the number of species) (Maurer and McGill 2011) based on the final densities of Collembola. Simpson evenness ranges from 0 to 1, values toward 1 mean higher similarity in abundances among species (Maurer and McGill 2011).

We then regressed changes in predator density as well as predator: prey ratio against experimental predator density treatments for the four plant communities separately in order to test how predator and prey densities changed during the experiment. Prey community evenness was also regressed against predator density in order to assess whether predator density caused prey community shifts. A linear mixed‐effect model (with Gaussian error) was used to incorporate blocks as the random effect. The model assumptions of homogeneity of variance were evaluated using the graphical observation of residual vs. fitted term graphs (increase in fitted terms had no effect on the residual terms, described in Zuur et al. 2009) and with Shapiro–Wilk test for the normality of residuals (*P* > 0.05). All models met the above assumptions and allowed parametric statistical tests except for the absolute prey density. The effects of predator density on the absolute prey density were evaluated using a generalized linear mixed model (GLMM) with a Poisson error. As absolute prey densities were count data (as opposed to predator density, which we expressed as the proportional change to indicate intraspecific competition), regression with Gaussian error was unable to account for heterogeneity and non‐negative nature of count data, which is why Poisson error was used for the regression analysis (Zuur et al. 2009).

We also used experimental predator density to explain variations in measured responses of the soil microbial community structure and biomass of key microbial functional groups, nitrification rates, ^15^N uptake by the plants, and plant biomass using a mixed‐effect linear regression with Gaussian errors and blocks as random effects. In cases where assumptions of parametric tests were not met with original data, log transformation of the response variables was performed (indicated in the Results text). The regressions were carried out for four plant communities separately. Plant shoot biomass, root biomass, and shoot biomass: root biomass ratio were regressed against predator density using linear mixed‐effect models. Shoot biomass per plant individuals from monocultures and the mixed plant community were analyzed without block effects using mixed linear regressions. Linear regression models were used to analyze the effects of predator density on complementarity and selection effects.

For microbial community analyses, principal component analysis (PCA) was carried out with 12 PLFA markers, which then resulted in PCA axis scores (1st axis was used) providing an index for microbial community composition. The PCA diagram from PLFA markers is provided as supplementary information (Appendix Figure S1).

Finally, we used a path analysis model for hypothesized relationships between the density of predators and plant complementarity and selection effects through shifts in prey community, soil microbial community composition, and nitrification rates. We used published hypotheses (Appendix Figure S2) and results of linear regressions to inform the initial path analysis model (Eisenhauer et al. 2015). The adequacy of path model was based on chi‐square tests and Akaike information criteria (AIC). We also tested direct relationships between predator density and plant complementarity and selection effects using linear regressions.

Both linear and generalized linear mixed‐effect models were run in R statistical software version 3.1.0 (R Core Team 2014) using “lme4” package (Bates et al. 2013). *P*‐values for mixed‐effect models were calculated from Wald chi‐square tests using the “car” package built for R statistical software (Fox et al. 2014). Further, marginal *R* ^2^ (for fixed effects only) and conditional *R* ^2^ (for combined fixed and random effects, i.e., block effects in this study) were calculated for each mixed‐effect models using the method explained in Nakagawa and Schielzeth (2013). Path model analysis was carried out in the “lavaan” package (Rosseel et al. 2013) built for R statistical software.

## Results

### Change in predator and prey densities

**Figure 1:**
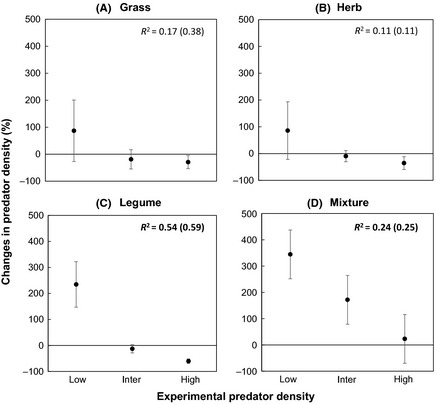
Experimental predator density effects on the final predator density expressed as the changes in predator density (in %) for four different plant communities using linear mixed‐effect models. Bold *R* ^2^ values represent significant relations. The *R* ^2^ values in brackets indicate conditional *R* ^2^ that combine variation explained by fixed (outside bracket *R* ^2^) and random effects.

Predator density significantly increased in low predator density treatments, whereas it declined in high predator density treatments in legume monocultures (*t*‐value = −3.99, *P*‐value < 0.0001, Fig. 1C) and the mixed plant community (*t*‐value = −2.13, *P*‐value = 0.03, Fig. 1D). We found a similar pattern of an increase in predator density in all low predator density treatments across all plant communities, although being statistically nonsignificant for grass monocultures (*t*‐value = −1.73, *P*‐value = 0.08) and herb monocultures (*t*‐value = −1.36, *P*‐value = 0.17). Interestingly, we found a relative increase in predator density at high predator density treatments only in the mixed plant community (Fig. 1 D, and Appendix Figure S3).

Realized predator: prey ratio also significantly decreased with the experimental predator density in legume monocultures (*t*‐value = −2.91, *P* < 0.01). However, grass monocultures (*t*‐value = 0.31, *P*‐value = 0.75), herb monocultures (*t*‐value = −0.53, *P*‐value = 0.59), and the mixed plant community (*t*‐value = 0.35, *P*‐value = 0.72) showed no significant changes in realized predator: prey ratio as affected by the experimental predator density (Appendix Figure S4).

**Figure 2:**
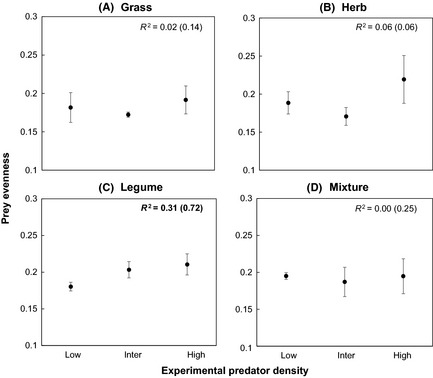
Prey evenness (Simpson evenness index) based on final prey density affected by experimental predator density. Bold *R* ^2^ stands for significant relations. The *R* ^2^ values in brackets indicate conditional *R* ^2^ that combine variation explained by fixed (outside bracket *R* ^2^) and random effects.

Prey evenness increased with predator density only in the legume monoculture (*t*‐value = 3.01, *P*‐value < 0.01). On contrary, prey evenness was unaffected by predator density in other plant communities (all *P*‐values > 0.05; Fig. 2). Absolute prey density was significantly affected by the predator density treatments. In grass monocultures, we found prey density to increase at high predator density treatments; however, we found a decline in prey density at low and intermediate predator densities (GLMM, *z*‐value = 2.90, *P*‐value < 0.01). Similar patterns were observed for herb (GLMM, *z*‐value = 3.48, *P*‐value < 0.001) and legume (GLMM, *z*‐value = 2.91, *P*‐value < 0.01) monocultures with higher prey densities at high predator density compared to low predator density treatments. These results show a relative prey suppression at low predator density, but an increase in prey density at high predator density (Appendix Figure S5). In contrast, prey density was lower only in the mixed plant community at intermediate and high predator density treatments (GLMM, *z*‐value = −6.91, *P*‐value < 0.0001) (Appendix Figure S5).

### Effects on microbial community

Effects of predator density on microbial community composition and biomass were inconsistent among plant communities (Table 1). However, we found a consistent decrease in the ratio between gram‐positive and gram‐negative bacteria across all plant communities (only legume monocultures showed a marginal decrease). Fungal biomass increased significantly with predator density in legume monocultures. Accordingly, the ratio between fungi and bacteria also increased with predator density in legume monocultures. The microbial community structure (PCA axis 1) was not strongly influenced by predator density, although a marginal effect was found in legume monocultures. Total PLFAs as an indicator of total living microbial biomass significantly decreased with predator density in the herb monoculture as well as in the mixed plant community (see Table 1 for details).

**Table 1:** Regression results based on linear mixed‐effect models for the response of microbial community groups (based on PLFA markers) to predator density for four plant communities (grass monoculture, herb monoculture, legume monoculture, and the mixed plant community). Bold letters indicate significant relationships (*P*‐value < 0.05). The direction of the relationship (increase/decrease) is shown by the sign in front of *t*‐values. The *R* ^2^ values in brackets indicate conditional *R* ^2^ that combine variation explained by fixed (outside bracket *R* ^2^) and random effects

PLFA markers	Grass			Herb			Legume			Mixture		
	*t*‐value	*P*‐value	*R* ^2^	*t*‐value	*P*‐value	*R* ^2^	*t*‐value	*P*‐value	*R* ^2^	*t*‐value	*P*‐value	*R* ^2^
Gram‐positive bacteria (GP)	−0.769	0.44	0.04 (0.05)	**−2.59**	**<0.01**	**0.32 (0.32)**	−1.5	0.13	0.13 (0.13)	**−2.54**	**0.01**	**0.33 (0.41)**
Gram‐negative bacteria (GN)	**2.24**	**0.02**	**0.21 (0.60)**	−1.38	0.16	0.10 (0.32)	0.1	0.91	0 (0)	0.15	0.87	0 (0)
GP: GN ratio	**−5.02**	**0.01**	**0.29 (0.29)**	**−2.33**	**0.01**	**0.21 (0.50)**	−1.57	0.11	0.15 (0.25)	**−2.28**	**0.02**	**0.23 (0.56)**
Fungi	0.35	0.71	0 (0.06)	−1.14	0.25	0.07 (0.30)	**2.62**	**<0.01**	**0.33 (0.41)**	0.38	0.69	0.01 (0.01)
Fungi: bacteria ratio	0.41	0.67	0.01 (0.13)	0.35	0.72	0 (0.30)	**2.82**	**<0.01**	**0.36 (0.36)**	1.58	0.11	0.14 (0.33)
Microbial community (PCA axis 1 scores)	−1.07	0.31	0.06 (0.06)	−0.28	0.77	0 (0)	−1.69	0.09	0.17 (0.17)	0.92	0.35	0.05 (0.05)
Total PLFA	−0.056	0.95	0 (0)	**−3.75**	**<0.001**	**0.5 (0.5)**	−0.111	0.91	0 (0)	**−2.34**	**0.01**	**0.28 (0.34)**

### Effects on nitrification rates

**Figure 3:**
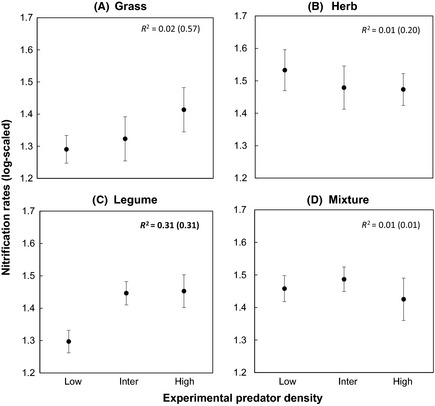
Variations in soil nitrification rates (log‐scaled) explained by increases in predator density. The *R* ^2^ values in brackets indicate conditional *R* ^2^ that combine variation explained by fixed (outside bracket *R* ^2^) and random effects obtained from linear mixed‐effect models.

Nitrification rates (log‐transformed) significantly increased with predator density in legume monocultures (*t*‐value = 2.51, *P*‐value = 0.01). Grass monocultures (*t*‐value = 0.47, *P*‐value = 0.63), herb monocultures (*t*‐value = −0.51, *P*‐value = 0.60), and the mixed plant community (*t*‐value = −0.47, *P*‐value = 0.63) showed no significant relation between nitrification rates and predator density (Fig. 3).

### Effects on plant performance

Plant shoot biomass showed a significant decline with predator density in grass and legume monocultures (Table 2). We also found a significant decline in the root biomass in grass and legume monocultures. A significant increase in shoot: root biomass ratio with predator density was found in legume monocultures only (Table 2). There was a consistent decline in shoot and root biomass in monocultures with increasing predator densities (being nonsignificant in herb monocultures though; Table 2), whereas no significant response in shoot and root biomass was observed in the mixed plant community.

**Table 2:** Plant community biomass (g/microcosm) response to predator density for four plant communities (grass monoculture, herb monoculture, legume monoculture, and the mixed plant community). Bold letters indicate significant relationships (*P*‐value < 0.05). The direction of the relationship (increase/decrease) is shown by the sign in front of *t*‐values. The *R* ^2^ values in brackets indicate conditional *R* ^2^ that combine variation explained by fixed (outside bracket *R* ^2^) and random effects

Plant biomass (Community)	Grass			Herb			Legume			Mixture		
	*t*‐value	*P*‐value	*R* ^2^	*t*‐value	*P*‐value	*R* ^2^	*t*‐value	*P*‐value	*R* ^2^	*t*‐value	*P*‐value	*R* ^2^
Shoot biomass (S)	**−2.34**	**0.01**	**0.27 (0.46)**	−1.35	0.17	0.08 (0.45)	**−2.01**	**0.04**	**0.22 (0.22)**	0.92	0.35	0.05 (0.05)
Root biomass (R)	**−2.13**	**0.03**	**0.24 (0.24)**	−0.29	0.76	0 (0.01)	**−2.25**	**0.02**	**0.26 (0.26)**	1.01	0.3	0.06 (0.06)
S:R ratio	−0.76	0.44	0.03 (0.27)	0.52	0.6	0.01 (0.04)	**1.97**	**0.04**	**0.16 (0.67)**	‐0.89	0.39	0.05 (0.17)

**Figure 4:**
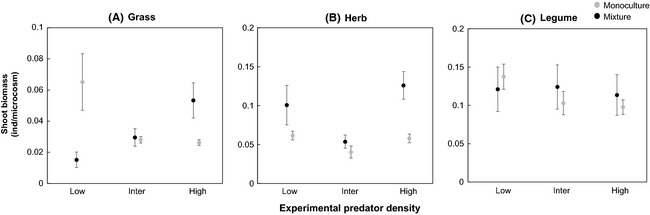
Variations in plant shoot biomass per plant individual per microcosm for monocultures and the mixed plant community explained by predator density. Linear regressions were used to obtain the relation (without random effects) shown in the figure.

Shoot biomass per grass individual was significantly affected by the interaction between plant community composition (monoculture versus mixed) and predator density (*t*‐value = −4.09, *P*‐value < 0.001; Fig. 4A). Grass shoot biomass increased in the mixed plant community, whereas it decreased in the monocultures with increasing predator density. Shoot biomass per herb individual was significantly different between the monoculture and the mixed community with higher biomass in the mixed plant community (*t*‐value = −1.19, *P*‐value < 0.01; Fig. 4B). Shoot biomass per legume individual was not significantly affected by predator density and plant composition.

The pattern of ^15^N uptake in plant species was analogous to shoot biomass per plant individual. ^15^N uptake in grass individuals was significantly affected by the interaction between plant community composition and predator density. That is, ^15^N uptake by the grass was higher at high predator density, but only in the mixed plant community (log‐transformed, *t*‐value = −3.90, *P*‐value < 0.001). ^15^N uptake in herb individuals was only affected by plant composition with higher uptake in the mixed plant community (*t*‐value = −1.68, *P*‐value = 0.01). We did not find any significant effect of predator density on ^15^N uptake in legumes in monoculture or in the mixed plant community.

**Figure 5:**
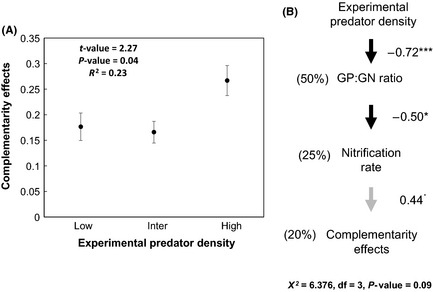
(A) Positive relationship between experimental predator densities and plant complementarity. (B) Path analysis model for the indirect relation between predator density and plant complementarity. In the path model diagram, gray arrows indicate positive relationships, whereas negative relationships are indicated by black arrows. The numbers in brackets denote explained variance expressed in percent for the causal relationships, whereas numbers adjacent to arrows represent standardized regression coefficients. The hypothesized path model is provided as supporting information together with the results of the path model on selection effects (Figure S2) (^**.**^ *P* = 0.05, *P < 0.05, ***P < 0.001).

Finally, a path model revealed that predator density explained variations in plant complementarity effects via shifts in soil microbial community composition (gram‐positive bacteria: gram‐negative bacteria ratio) and subsequently nitrification rates (Fig. 5B). Predator density significantly decreased gram‐positive bacteria: gram‐negative bacteria ratio (path coefficient = 0.72, *P*‐value < 0.001) explaining 50% of the variance in the proxy of soil microbial community composition. Subsequently, a decline in the ratio between gram‐positive bacteria and gram‐negative bacteria was negatively correlated to nitrification rates (path coefficient = −0.50, *P* = 0.03). Changes in nitrification rates were then positively associated with the plant complementarity effect (path coefficient = 0.44, *P*‐value = 0.05) and explained 20% of the variation in the complementarity effect (Fig. 5B). In contrast to the cascading effects of predator effects on plant complementarity, we did not find any significant relation for the selection effect. The observed indirect positive relation between predator density and complementarity effects were supported by the observed positive relation between experimental predator density and plant complementarity effects from linear regression (Fig. 5A). We also did not find any significant association between experimental predator density and the selection effect (*t*‐value = −0.19, *P*‐value = 0.84). The path model with a direct path between prey community shift and the ratio between gram‐positive and gram‐negative bacteria was nonsignificant (path coefficient = 0.20, *P* > 0.05, Appendix Figure S2).

## Discussion

Our results provide an experimental evidence for density‐mediated cascading effects of belowground predators on plant performance through shifts in soil microbial communities and a crucial part of N dynamics in soil (nitrification rates). The strength and direction of these cascading effects varied among plant species of different functional groups. Our findings indicate that density effects of predators could differentially affect plant performance in monoculture compared to their performances in mixed plant communities. We argue that such variations are potentially due to differences in cascading effects of predator density on soil microbial community composition, nitrification rates, and plant uptake of soil N. Importantly, this caused a positive association between experimental predator density and plant complementarity. Thus, some plant community shifts may only be mechanistically understood if roles of distant trophic groups, such as belowground predators, on nutrient cycling are considered (Schmitz et al. 2010).

### Change in predator and prey densities

A consistent increase of predator population size in the low predator density treatments across plant communities indicates a positive density dependence, that is, a positive intrinsic growth rate of predators. Studies have shown that positive density dependence occurs when individuals of species experience minimal intraspecific competition and are distant from their carrying capacity (Courchamp et al. 1999). We found a pattern of convergence in predator population densities at the end of the experiment for all density treatments, although mainly for plant monocultures and most pronounced in the legume monoculture (Appendix Figure S3). This implies that high‐density treatments of predators decreased in density at the end of the experiment likely due to higher intraspecific competition in the form of interference or potential cannibalism at limited prey availability (Polis 1981), a phenomenon that has been reported for predatory mites (Berndt et al. 2003). Furthermore, such changes in predator population concur with the observed suppression of prey density and shifts in prey community composition, particularly in plant monocultures and at low predator density (Fig. 2, Appendix Figure S5). Decreased prey evenness at low predator density indicate that increases in predator population trigger higher variation of density among prey species (Filip et al. 2014). Indeed, studies have shown that predator‐induced prey suppression can induce compensatory population growth in prey species of faster regeneration (Filip et al. 2014; Thakur and Eisenhauer 2015), which may decrease prey evenness.

### Shifts in soil microbial community and nitrification rates

Two contrasting patterns emerged among soil microbial communities in response to predator density effects. First, two clear trophic cascades occurred: an increase in fungal biomass in the legume monoculture and an increase in gram‐negative bacterial biomass in the grass monoculture (Table 2). Second, the biomass of gram‐positive bacteria decreased in the herb monoculture and the mixed plant community with increasing predator density. Positive effects of predators on soil microbial communities have been demonstrated in other studies (reviewed in Wardle 2002). Although our results contradict studies that have reported no evidence of soil predator effects on microbial communities (Mikola and Setälä 1998; Sackett et al. 2010), we further show that trophic cascades in soil are context dependent and differ between plant communities. For instance, predator‐induced suppression of Collembola favored fungi over bacteria in the legume monoculture, which are often associated with higher bacterial and fungal biomass in soil than nonleguminous plant species (Chen et al. 2008). Community shifts of Collembola and plant community‐specific substrate environments (such as in the form of differences in the quantity and quality of rhizodeposition) could interactively affect the soil microbial community. Fungi, for instance, often benefit more from high substrate availability than bacterial communities do (Griffiths et al. 1999) and from reduced grazing pressure (Crowther et al. 2011).

Nitrification has been shown to increase in the presence of higher densities of microbial grazing Collembola, particularly due to the stimulation of nitrifying bacteria (Cragg and Bardgett 2001). In the present study, nitrification rates were significantly lower in low predator density treatments in legume monocultures, where Collembola suppression was high with a lower community evenness (Fig. 2C). It is important to note that nitrification rates principally increase at higher ammonium availability with a subsequent reduction in ammonium immobilization by soil microorganisms, among other factors (Roberston and Groffman 2007). Importantly, as legume species are inferior at acquiring ammonium from soil compared to nonleguminous species (Von Felten et al. 2009), it is possible that microbial immobilization of ammonium would increase when Collembola‐induced microbial turnover is low (Bardgett and Chan 1999).

### Plant community responses

We did not find any contrasting responses between legume and nonleguminous plant monocultures in terms of their community biomass as affected by predator density, contradicting our hypothesis 2 (Table 2). We observed a consistent decrease in shoot and root biomass at high predator density in both legume and grass monocultures. The decrease in shoot and root biomass of legume monocultures despite an increase in nitrification rates indicates a poor performance of legume species at higher availability of nutrients (Jackson et al. 2008). This, however, may vary among legume species (Fan et al. 2002), although particularly *Trifolium* species have been shown to have lower nitrate uptake rates compared to grass and herb species (Näsholm et al. 2000). Moreover, previous studies have shown that higher availability of nitrate in soil can impair root nodulation in legumes and suppress N fixation (Becana and Sprent 1987), thereby reducing legume biomass (Naudin et al. 2011), which goes along with increased nitrification rates. The poor performance of grass monocultures at high Collembola density (i.e., at high predator density) is somewhat surprising as grasses typically perform well at elevated nutrient availability (Hodge and Fitter 2012). However, our finding resembles with studies reporting decreased root biomass of a grass monoculture in the presence of Collembola (Scheu et al. 1999; Sabais et al. 2012). The authors of these studies speculate that Collembola might selectively feed on grass roots, which may surpass the advantages from nutrient mineralization.

Individual plant shoot biomass showed a contrasting pattern in monocultures as compared to the mixed plant community. Particularly grass individuals produced more biomass in the mixed plant community than in monoculture (Fig. 4A). A previous study reported that grasses performed better than legumes in grass–legume mixtures at increased N availability in soil due to the presence of detritivores (Eisenhauer and Scheu 2008). Our results of enhanced performance of the grass species in the presence of the legume confirmed our hypothesis 3 and could be due to enhanced N availability in soil. This might have been most pronounced at high predator density, although we did not find any clear trend of nitrification rates in the mixed plant community (Fig. 3D). Furthermore, higher ^15^N uptake in the grass species (indicating N uptake from soil) in the mixed plant community than in monoculture indicates that grass individuals had a competitive advantage in the presence of the legume species.

Our path model confirmed that predator density can cause indirect effects on plant complementarity via shifting microbial community composition (ratio between gram‐positive and gram‐negative bacteria) and subsequent effects on the nitrification rate (Fig. 5B). The negative correlation between predator density and gram‐positive: gram‐negative bacteria ratio indicates a compositional shift in the microbial community (Fierer et al. 2003). Such soil microbial compositional shifts may occur due to variability in the substrate availability (Williams and Rice 2007; Montaño et al. 2009). This could affect the nitrification process due to subtle differences in substrate use between gram‐positive and gram‐negative bacteria (Waldrop et al. 2000). For instance, gram‐positive bacteria are less efficient in converting organic substrates from plant roots than gram‐negative bacteria (Bird et al. 2011). Based on our path model results, we speculate that a decrease in nitrification rates with changes in the ratio between gram‐positive and gram‐negative bacteria could be attributed to a reduced potential of soil microorganisms in utilizing available substrates. However, we stress that the mechanistic understanding of shifts in the microbial community and associated variation in nitrification rates would require identification of nitrifying microbial species and their specific associations with the plant community.

Positive effects of experimental predator density on plant complementarity effects confirm that predator density could alter interactions among plant species. Notably, an increase in complementarity results from a reduced interspecific competition among plant individuals in the mixed community compared to intraspecific competition in monocultures (Loreau and Hector 2001). Nonleguminous plants can benefit from neighboring legume species by receiving legume‐derived N (Temperton et al. 2007), and predators might have re‐enforced such benefits to grass individuals in the present study. The improved performance of grass individuals at high predator density treatments in the mixed plant community compared to the monoculture provides support for this speculation. Interestingly, several studies have highlighted the role of plant complementarity in mixed plant communities causing increased productivity at high plant diversity (Loreau and Hector 2001; Cardinale et al. 2007; Isbell et al. 2009). Our results provide some evidence of the potential role of multitrophic interactions in soil affecting plant complementarity and hence the plant diversity–productivity relationships (Duffy 2002).

## Conclusion

Our study provides empirical evidence of density‐dependent cascading effects of belowground predators of detritivores on soil microbial community composition, soil nitrification rates, and plant N uptake with the strength and direction of effects depending on plant identity and community composition. Further, we found altered plant performance and competition due to belowground predators. The distinct effects of predator density on soil processes and plant complementarity suggest that the simplification of soil food webs due to land‐use intensification and climate change can significantly alter aboveground–belowground interactions and associated ecosystem functions.

## Conflict of Interest

None declared.

## Supporting information


**Figure S1.** PCA diagram for PLFA markers. The suffix indicate microbial functional groups (GP = gram‐positive bacteria, GN = gram‐negative bacteria and Fu = fungi).
**Figure S2. (**Left panel): Conceptual diagram showing the hypothetical relations based on the literature for the path analysis. The gray arrows indicate that a variable could influence change in the other variable. We do not explicitly show direction of the influence due to mixed results reported in the literature. (Right panel): The path analysis results for the relation between predator density and plant complementarity with inclusion of prey evenness (Details in the main text).
**Figure S3.** Observed patterns of density changes of predators during the experiment (two time points).
**Figure S4**. Experimental predator density effects on the predator: prey ratio at the end of the experiment for four plant communities.
**Figure S5.** Effects of predator density treatments on prey density at the end of the experiment.
