# Supplementary material for: Cascading effects of belowground predators on plant communities are density‐dependent
Source: Ecol Evol. 2015 Sep 12;5(19):4300–14. doi: 10.1002/ece3.1597 (PMC4667818; doi:10.1002/ece3.1597)
Supplement: Supplementary file 1 — Figure S1. PCA diagram for PLFA markers. The suffix indicate microbial functional groups (GP = gram‐positive bacteria, GN = gram‐negative bacteria and Fu = fungi). Figure S2. (Left panel): Conceptual diagram showing the hypothetical relations based on the literature for the path analysis. The gray arrows indicate that a variable could influence change in the other variable. We do not explicitly show direction of the influence due to mixed results reported in the literature. (Right panel): The path analysis results for the relation between predator density and plant complementarity with inclusion of prey evenness (Details in the main text). Figure S3. Observed patterns of density changes of predators during the experiment (two time points). Figure S4. Experimental predator density effects on the predator: prey ratio at the end of the experiment for four plant communities. Figure S5. Effects of predator density treatments on prey density at the end of the experiment. [file ECE3-5-4300-s001.pdf]

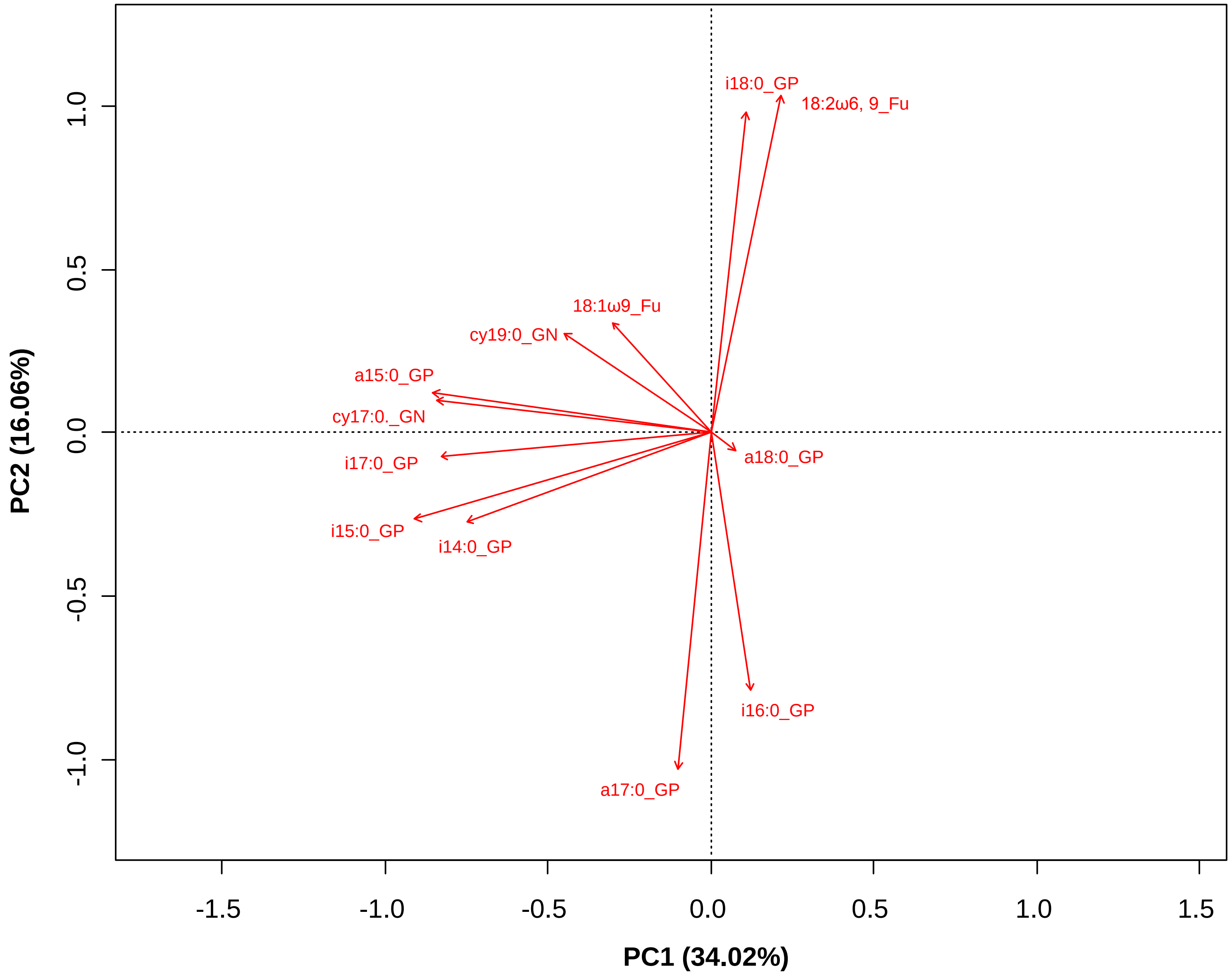

Figure S1: PCA diagram for PLFA markers. The suffix indicate microbial functional groups (GP= gram- positive bacteria, GN= gram-negative bacteria and Fu= fungi)

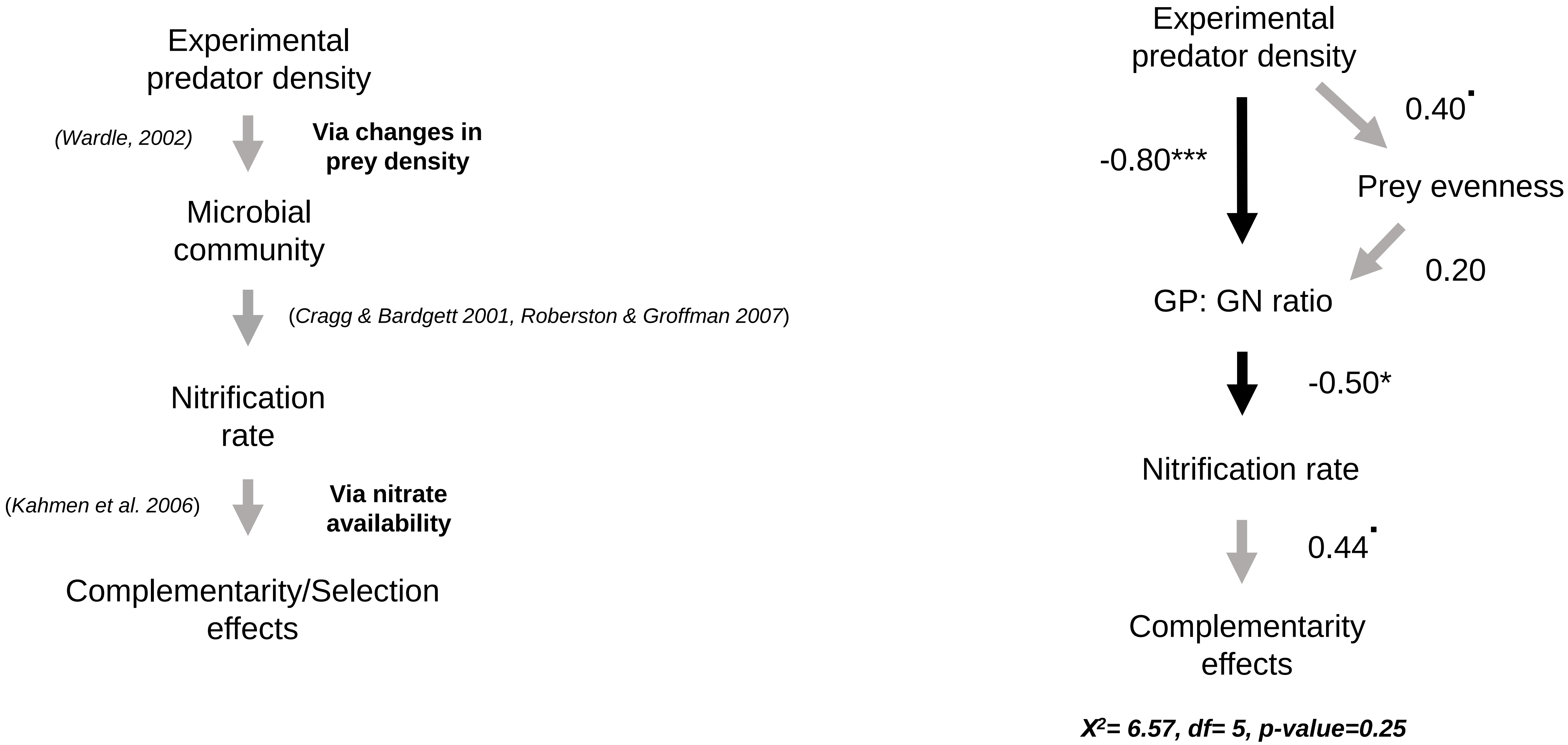

Figure S2 (Left panel): Conceptual diagram showing the hypothetical relations based on the literature for the path analysis. The gray arrows indicate that a variable could influence change in the other variable. We do not explicitly show direction of the influence due to mixed results reported in the literature. (Right panel): The path analysis results for the relation between predator density and plant complementarity with inclusion of prey evenness (Details in the main text). Reference details are provided at the end of the Appendix.

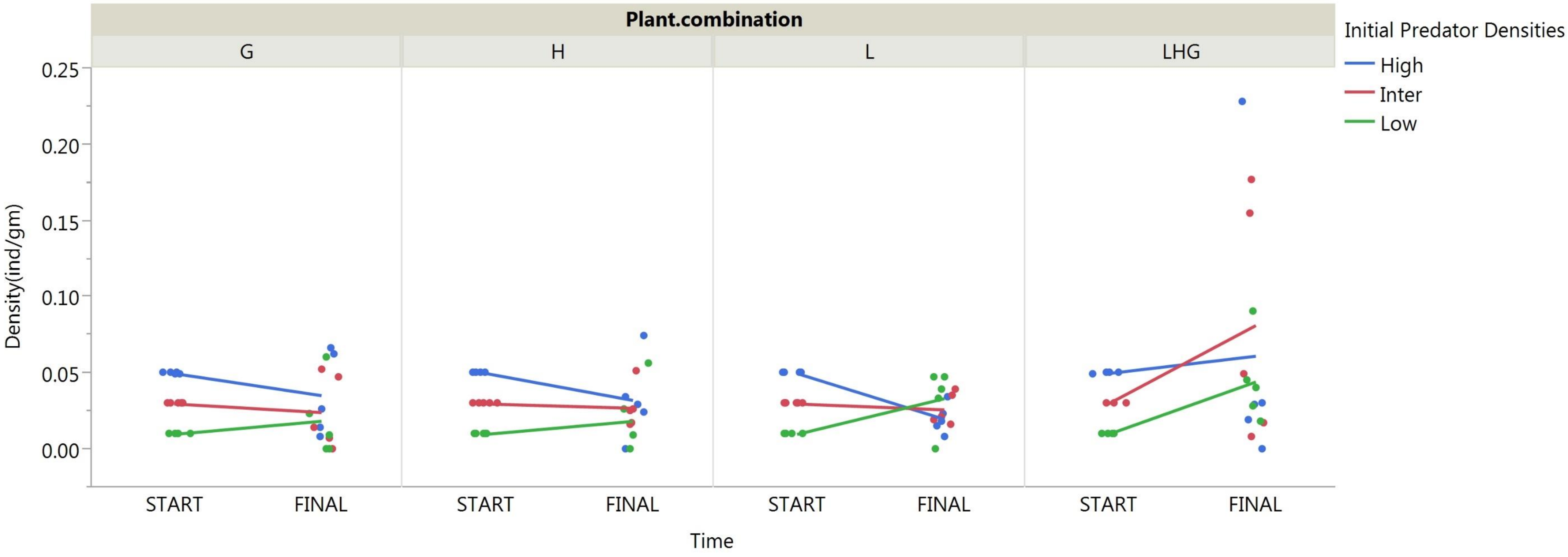

Figure S3: Observed patterns of density changes of predators during the experiment (two time points). Predator densities in the monoculture communities show a converging patterns whereas in the mixed community, we observed an overall increasing trend. We found a significant interaction effect between predator density and two time points in the legume monoculture ($p<0.01$) indicating increase of predator density in low density treatments whereas decline in density in high density treatments. G=Grass monoculture, H= Herb monoculture, L= Legume monoculture, LHG = Mixed plant community.

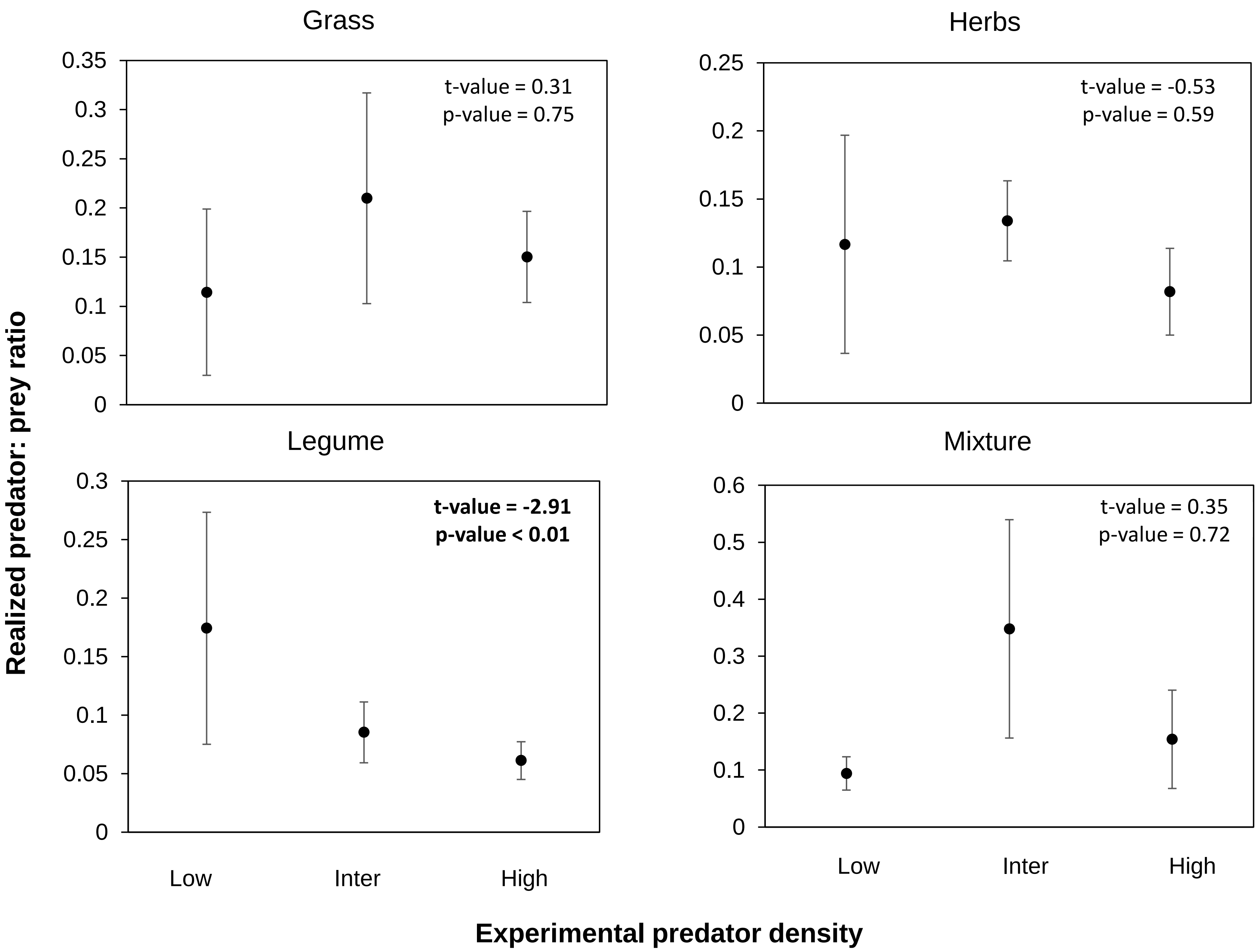

Figure S4: Experimental predator density effects on the predator: prey ratio at the end of the experiment for four plant communities. Bold R^2 values represent significant relation
The R^2 values inside bracket indicate conditional R^2 that combines variation explained by fixed (outside bracket R^2) and random effects.

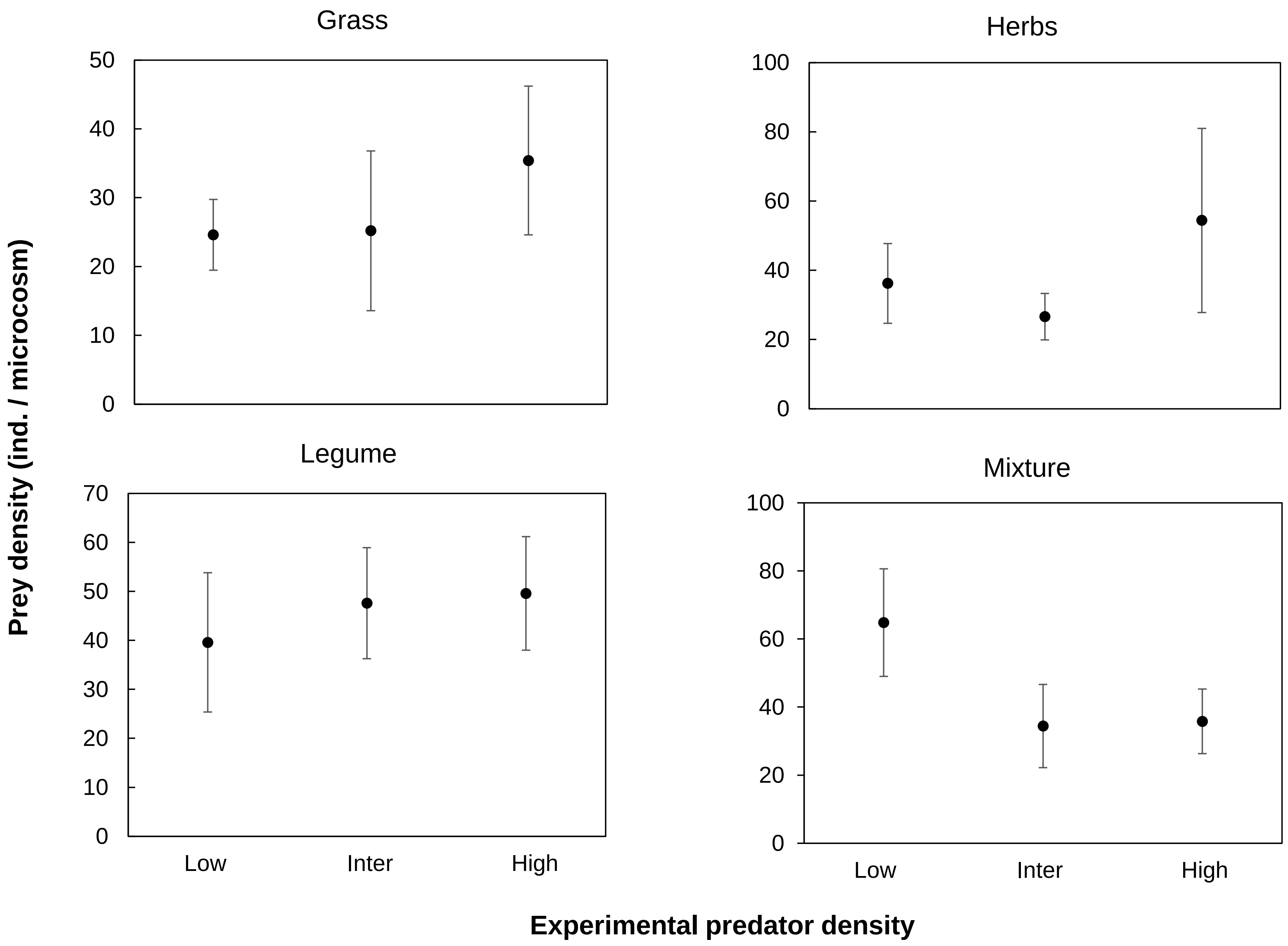

Figure S5: Effects of predator density treatments on prey density at the end of the experiment. We found a relative suppression (Details of statistical analyses are provided in the main text) of prey at the low predator density treatments except for the mixed plant community.

References

Wardle, D.A. (2002) *Communities and Ecosystems: Linking the Aboveground and Belowground Components*. Princeton Univ Press.

Cragg, R. & Bardgett, R. (2001) How changes in soil faunal diversity and composition within a trophic group influence decomposition processes. *Soil Biology and Biochemistry*, **33**, 2073–2081.

Roberston, G.. & Groffman, P.M. (2007) Nitrogen transformations. *Soil microbiology and biochemistry* (ed E.A. Paul), pp. 341–362. Elsevier Academic Press.

Kahmen, A., Renker, C., Unsicker, S. & Buchmann, N. (2006) Niche complementarity for nitrogen: an explanation for the biodiversity and ecosystem functioning relationship? *Ecology*, **87**, 1244–1255.
